# S100A4 in the Physiology and Pathology of the Central and Peripheral Nervous System

**DOI:** 10.3390/cells10040798

**Published:** 2021-04-02

**Authors:** Nadia D’Ambrosi, Martina Milani, Savina Apolloni

**Affiliations:** Department of Biology, University of Rome Tor Vergata, 00133 Rome, Italy; martina1992.mm@gmail.com

**Keywords:** astrocytes, microglia, neurodegeneration, neuroinflammation, brain tumors

## Abstract

S100A4 is a member of the large family of S100 proteins, exerting a broad range of intracellular and extracellular functions that vary upon different cellular contexts. While S100A4 has long been implicated mainly in tumorigenesis and metastatization, mounting evidence shows that S100A4 is a key player in promoting pro-inflammatory phenotypes and organ pro-fibrotic pathways in the liver, kidney, lung, heart, tendons, and synovial tissues. Regarding the nervous system, there is still limited information concerning S100A4 presence and function. It was observed that S100A4 exerts physiological roles contributing to neurogenesis, cellular motility and chemotaxis, cell differentiation, and cell-to cell communication. Furthermore, S100A4 is likely to participate to numerous pathological processes of the nervous system by affecting the functions of astrocytes, microglia, infiltrating cells and neurons and thereby modulating inflammation and immune reactions, fibrosis as well as neuronal plasticity and survival. This review summarizes the current state of knowledge concerning the localization, deregulation, and possible functions of S100A4 in the physiology of the central and peripheral nervous system. Furthermore, we highlight S100A4 as a gene involved in the pathogenesis of neurological disorders such as brain tumors, neurodegenerative diseases, and acute injuries.

## 1. Introduction

**Figure 1:**
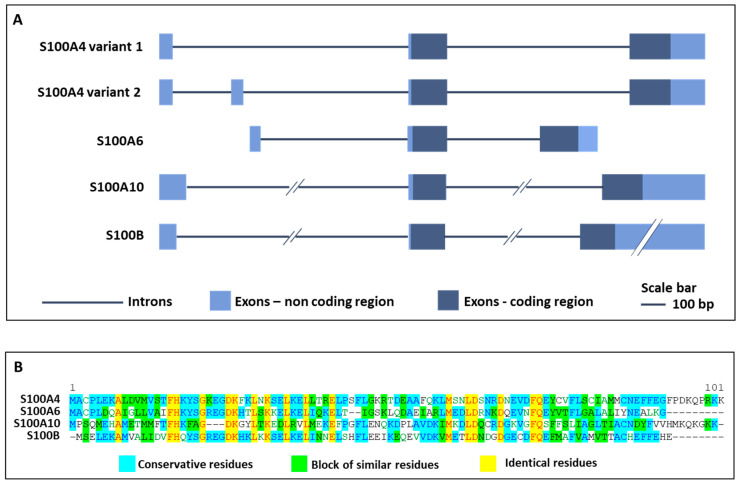
Exon-intron organization and protein sequence comparison of S100A4, S100A6, S100A10 and S100B. The figure shows the exon-intron organization (**A**) and the protein sequence comparison (**B**) of S100A4, S100A6, S100A10 and S100B, S100 family members mainly involved in nervous system diseases.

S100A4, also called fibroblast-specific protein 1 (Fsp1) or metastasin 1 (MTS1), is a member of the large family of S100 proteins, containing two EF-hand Ca^2+^-binding motifs, and with a common function of sensing and responding to variations of intracellular calcium concentrations [1,2,3]. S100A4 is predominantly localized in the cytoplasm of an extensive variety of cell types, although several studies identified its presence in the nucleus upon defined post-translational modifications or stimuli [4,5]. In addition, S100A4 can be secreted in the extracellular space to exert autocrine and paracrine actions [6]. The human S100A4 gene resides in chromosome 1q21 and comprises two isoforms with three or four exons that code for a protein with 101 amino acid residues (Figure 1A,B). The functional S100A4 is a polypeptide assembled as an antiparallel homodimer of 22 kDa, able to oligomerize upon defined Ca^2+^ concentrations and reducing conditions, and to change its conformation upon alterations in intracellular Ca^2+^ levels, thereby modifying the interaction with target proteins and leading to changes in their activity [7,8]. Indeed, S100A4 regulates the cytoskeleton organization by directly interacting with proteins such as non-muscle myosin-IIA, tropomyosin, liprin β1, and ezrin, with consequent changes in cell morphology, adhesion and migration [9,10]. S100A4 has been considered for a long time as a protein involved only in tumorigenesis, but over the last few years, it was involved in several fibrotic and inflammatory processes [9,11]. Initial functions attributed to S100A4 were to support metastatization, given its ability to bind and regulate cytoskeletal components, thereby promoting the motility and invasion of several types of tumor cells. Moreover, S100A4 is considered a metastasis-promoting factor also because the protein is released by activated stromal cells (e.g., fibroblasts, immune and vascular cells) in the tumor microenvironment, where it fosters growth factors release, angiogenesis and overall tumor survival. The increased protein secretion associated with tumor development and invasiveness has made S100A4 a good biomarker for different metastatic cancers, where it also assumes a prognostic value, since its increase is associated with poor patients survival [12,13,14,15,16]. Besides these roles, S100A4 controls different cellular pathways, exerting in this way numerous effects on processes that are cell- and tissue-type dependent. In activated fibroblasts, endothelial, dendritic and mast cells as well as in macrophages, monocytes, neutrophils, and T-lymphocytes, S100A4 has a significant role in stimulating invasion and migration, cytoskeletal dynamics and in promoting pro-inflammatory phenotypes. Li and coauthors demonstrated that mice lacking S100A4 showed impairment in macrophage recruitment to the sites of inflammation, and that primary macrophages derived from these mice exhibited defects in chemotaxis and alterations in matrix-degrading ability [17]. When secreted, S100A4 can recruit immune cells, stimulate pro-inflammatory pathways and lead to the secretion of cytokines and extracellular matrix (ECM) remodeling proteins [8]. The mechanism of S100A4 secretion occurs in a non-canonical way, likely via lysosome secretion, direct protein export, multivesicular body release or plasma membrane blebbing. When in the outer environment, S100A4 forms oligomers that can interact with several cell surface receptors, as the receptor for advanced glycation end products (RAGE), common to the S100 members, Toll-like receptor 4 (TLR4), epidermal growth factor receptor (EGFR), IL-10 receptor (IL10R), and annexin-A2 [9].

S100A4 is now emerging as an important player of organ fibrosis, such as in liver, kidney, lung, heart, as well as in tendons and synovial tissues, by promoting fibroblast activation, ECM remodeling and inflammatory cell recruitment [11]. In fact, S100A4 represents a well-known marker that characterizes a complex biological process where endothelial cells assume a mesenchymal phenotype, known as the “endothelial-to mesenchymal transition”, changing morphology and functions and acquiring accentuated motility and contractile properties, typical of fibrotic processes.

Although it is amply reported that S100A4, acting both intra- and extracellularly, regulates cell functions in many tissues such as muscle, lung, bone and intestine, there is still limited information concerning its presence and function in the nervous system. Indeed, despite having been demonstrated that at least 12 members of the S100 family, including S100A4, are expressed in the healthy and injured nervous system, and in particular S100B [18], S100A6 [19], and S100A10 [20] (Figure 1A,B), the signaling pathways of S100A4 in the physiopathology of this tissue is yet to be fully elucidated. This review aims to summarize the role of S100A4 in different physiological and pathological conditions of the nervous system and highlight S100A4 as a common factor involved in the pathogenesis of neurological diseases.

## 2. S100A4 in the Nervous System Physiology

S100A4 is expressed in both the central (CNS) and peripheral nervous system (PNS), where it is found in glial cells and satellite cells, as well as in neurons, in particular in cell subpopulations belonging to dorsal root ganglia (DRG), sympathetic neurons, trigeminal, geniculate and nodose ganglion cells [21,22,23,24,25]. Spatially, the presence of S100A4 in the rodent nervous system has been detected mainly in CNS myelinated areas as the olfactory tract, optic nerve, corpus callosum, internal capsule, fimbria, and spinal cord funiculi. However, the protein was also found in several nonmyelinated or poorly myelinated areas, such as the pituitary gland, the olfactory bulb and Lissauer’s tract [22,24].

Different indications suggest that S100A4 contributes to the development of the nervous system. It was demonstrated that in the human hippocampus and temporal cortex, S100A4 displayed a specific spatio-temporal pattern of prenatal expression, characterized by decline in aging [26]. In addition, S100A4 immunoreactivity postnatally appeared in newly myelinated areas, advocating a role of the protein in the maturation of myelinated fiber tracts [22,27]. Moreover, S100A4 is also related to the aging process since in the human brain the protein, with other S100 family members, was found in corpora amylacea, hyaline structures associated with the normal aging, suggesting its function in senescence-associated inflammation [28].

Like in other tissues, in the nervous system, S100A4 appears as a multifunctional protein with intracellular as well as extracellular functions, able to regulate neuronal survival and plasticity and responses to injury and disease.

### 2.1. Neuronal Cells

Although only a few populations of neurons express S100A4 [21,22,23,24], many types of neuronal cells can instead respond to extracellular S100A4, as they possess different receptor types or interactors capable of binding S100A4. The possible effects of S100A4 on neuron survival and neurite outgrowth were assessed in different primary neuronal cultures and with several experimental paradigms, ranging from direct exogenous S100A4 administration, to neuron-astrocytes co-cultures. Exposure to S100A4 promoted neurite outgrowth and survival after apoptotic stimuli in hippocampal, dopaminergic and cerebellar neurons, in a RAGE-independent fashion [29,30]. These effects, mediated by extracellular S100A4, mainly occurred when the protein was arranged in oligomeric quaternary structures and interacted with heparan sulfate proteoglycans at the surface of neuronal cells [29,30], leading to Ca^2+^ entry via nonselective and T-/L-type voltage-gated Ca^2+^ channels [31]. These results led to the hypothesis that S100A4, possibly released by astrocytes, could exert trophic effects on neurons. However, this theory was only partially confirmed in experiments employing DRG neurons co-cultured with white matter (WM)-derived S100A4+-astrocytes. In this circumstance, the decrease of S100A4 expression in astrocytes promoted neurite growth, rather than inhibiting it, while the application of recombinant S100A4 on the co-cultures induced extensive growth of DRG neurites [32]. In contrast, overexpression of S100A4 in Schwann cells co-cultured with spiral ganglion neurons increased the levels of neurite-developing marker GAP43, while S100A4 knockdown decreased nerve growth [33]. Interestingly, the reported actions suggest that the effects of S100A4 on neurons can be cell-type specific, can depend on the amount of the extracellular protein, on its quaternary structure and on the local environment provided by the presence of additional cell types.

### 2.2. Neuroglial Cells

One of the main cell components of the CNS expressing S100A4 in healthy conditions is represented by astrocytes and in particular, by those residing exclusively in the spinal cord WM [21,34] and preferentially localized in the subpial regions of the mature spinal cord [27]. However, upon different types of injury, S100A4 dramatically increases its expression in both white [21,35] and gray matter [34], evidencing a role for the protein in damaging o reparative mechanisms associated with tissue impairment.

The expression of S100A4 in astrocytes in culture influenced cytoskeletal reorganization, affecting their motility. Takenaga and Kozlova demonstrated that contrary to what occurs in other cell types, as cancer and immune cells [36], S100A4 was an inhibitor of WM astrocytes motility since its silencing promoted their migration and the expression of the metalloproteinase (MMP)-9 and metallothionein-1. In addition, the inhibition of MMPs activity significantly reduced the migration of S100A4-depleted astrocytes, suggesting that S100A4, affecting MMPs activity, provided stabilizing properties to WM astrocytes that could contribute to the formation of a rigid, growth-inhibitory glial scar [37]. The reduced motile ability associated with S100A4 expression in WM astrocytes was further confirmed in the in vitro injury model of the scratch assay [38]. However, in Schwann cells in vitro S100A4 exerted opposite functions on motility, with respect to WM astrocytes. Indeed, the migration of Schwann cells was significantly enhanced by S100A4 overexpression and was accordingly suppressed by S100A4 knockdown. The migratory phenotype obtained from S100A4 overexpression was accompanied by an increase of molecules promoting cell migration as vascular endothelial-derived growth factor and MMP-9 [33]. Therefore, the phenotype associated with S100A4 expression in glial cells is not univocal and, established that the protein is involved in cell motility, its inhibitory or stimulatory effect on cell migration could depend on the cell type. Besides the influence on cell motility, another function that has been ascribed to S100A4, and in particular to the extracellularly released protein, was to drive tunneling nanotubes (TNTs) direction between astrocytes and between astrocytes and neurons. TNTs are structures responsible for transferring cellular contents from healthy to injured cells and S100A4, putatively through its receptor RAGE, was shown to be involved in forming a gradient guiding the growth direction of TNTs [39].

In healthy conditions, microglia, the resident immune cells of the CNS, expressed low levels of S100A4, but the amount of the molecule increased on injury [34,40] or following activation in vitro with different pro-inflammatory stimuli, as tumor necrosis factor-α, ATP and lipopolysaccharide [34]. Remarkably, the S100A4 transcriptional inhibitor niclosamide, prevented NADPH oxidase 2, mammalian target of rapamycin (mTOR), and nuclear factor-κB (NF-κB) increase in reactive cells. In addition, the inhibition of S100A4 affected cytoskeletal rearrangements, migration and phagocytosis [34], suggesting that S100A4 in microglia possessed pro-inflammatory functions, in analogy to what occurs in peripheral immune cells [41]. It can therefore be speculated that S100A4 can trigger the activation of pro-inflammatory pathways and enhance the motility and phagocytic activity of reactive cells, presumably to achieve sites of injury and clear unwanted material.

## 3. S100A4 in the Pathology of the Nervous System

Like other members of the S100 family, S100A4 protein is involved in different pathologic states affecting the nervous system. While it is now clear that in several types of insults in the CNS and PNS the expression of S100A4 is often dramatically increased, the function of the protein in the injured nervous system is still poorly characterized and does not appear so obvious. Within the next paragraphs, we discuss the expression and function of S100A4 in different type of diseases affecting the nervous system.

### 3.1. Brain Tumors

S100A4 takes part in many aspects of tumor progression and invasiveness, such as the control of cell cycle, angiogenesis, cell adhesion and motility. Indeed, S100A4 is a recognized interactor of the tumor suppressor p53, promoting its degradation and progression into the cell cycle, which may result in tumor development [42]. In addition, intracellular and extracellular S100A4 are implicated in several events characterizing the complex process of metastatization [11]. Accordingly, the inhibition of S100A4 expression in tumor cells suppresses their metastatic potential, and represents a strategy to counteract metastatic cancers. In this regard, the S100A4 inhibitor niclosamide is currently in phase II clinical trial for metastatic colorectal cancer (NCT02519582) and prostate cancer (NCT02807805) and has completed phase I trial for prostate cancer (NCT02532114) [43,44,45]. Furthermore, an increase of S100A4 release in biological fluids was observed in different types of cancers such as brain tumors, breast, colon and lung carcinomas, demonstrating a possible exploitation of S100A4 as a prognostic biomarker to detect early stage tumors and as a marker to evaluate metastatic events [9]. S100A4 characterizes the properties of different brain tumors, representing both a promising biomarker and a potential target for their demise [46].

The most common malignant brain tumor in children, medulloblastoma, displays up-regulated levels of S100A4 [47]. In the medulloblastoma cell line Daoy cells, Erb-B2 Receptor Tyrosine Kinase 2 (ERBB2) overexpression, an event associated with invasiveness and poor prognosis, increased the migration across basement membranes in vitro and the expression of prometastatic genes, such as S100A4. The latter was demonstrated to be a direct target of ERBB2 signaling through a pathway involving protein kinase B (AKT), phosphoinositide 3-kinases (PI3K), and extracellular signal-regulated kinase (ERK1/2). The levels of ERBB2 and S100A4 tightly correlated also in samples of primary medulloblastoma [48].

Like medulloblastoma, ependymoma is one of the most frequent brain tumors in infants, with around 50% occurring in children above five years of age. Gain of 1q is a frequent event in ependymoma, occurring at an incidence of more than 20%. Using a combination of comparative genome hybridization and serial analysis of gene expression, candidate genes on 1q were identified, validated by immunohistochemistry and the protein expression levels correlated with clinicopathological data to determine their potential role. S100A4 was found as one of the most up-regulated genes in tumor samples, and its expression strongly correlated with patients aged less than three years at diagnosis of intracranial ependymoma [49].

Glioblastoma multiforme (GBM) is the most represented primary tumor of the CNS in adults and arises from glial or precursor cells. Based on histopathological and clinical features, it was classified as being in the highest grade of malignant tumors, and patients affected by GBM have poor prognosis. Epigenetic alterations, such as DNA methylation, have emerged as a common hallmark of human cancer, including GBM. Methylation patterns in DNA sequences associated with hypomethylated oncogenes and hypermethylated suppressors are important features that mediate carcinogenesis and correlate significantly with patient survival [50]. A recent study obtained from integrated analyses of methylated-differentially expressed genes in primary GBM revealed that S100A4 was one of the most significant hypomethylated/overexpressed genes characterizing the tumor, and that its elevated levels were associated with poor overall survival in GBM patients [51]. High levels of S100A4 appeared typical of high-grade GBM, as low-grade astrocytic tumors displayed lower S100A4 content [52]. These results identified S100A4 as a specific and sensitive diagnostic and prognostic biomarker in GBM and as a possible therapeutic target. Concerning the latter aspect, Takenaga and colleagues showed that S100A4 down-regulation could influence tumor migration both in vitro and in vivo, suggesting that the protein was involved in the motility and invasiveness of GBM. It was established that in vitro, the migration of C6 glioma cells depended on the expression of S100A4, as its silencing inhibited their motility [52]. In addition, the direct extracellular administration of S100A4 to astrocytic tumors modified their cytoskeletal arrangement and stimulated their migration rate [53], demonstrating a role also for the secreted protein in the migratory phenotype. In this aspect, it was demonstrated that the expression of S100A4 in co-cultured astrocytes surrounding C6 cells could positively correlate with tumor motility. These findings demonstrated that the expression and release of S100A4 drive glioma cells migration [52].

Finally, S100A4 was one of the highly expressed genes in meningioma, a common primary tumor of the CNS that originates from the arachnoid. Several data reported that there are sex differences in the incidence and aggressiveness of this type of tumor, with females being more affected than males, but with the latter usually developing a more aggressive form. By the use of shotgun proteomics, comparing the profile of grade I meningioma biopsies of male and female patients, a significant differential expression of several proteins was revealed between the two groups, with a higher abundance of cell-matrix organization genes as S100A4 in males, and thus suggesting that the protein was related to the higher cancer aggressiveness and poorer prognosis of patients [54].

### 3.2. Neurodegenerative Disorders

Regardless of the continuous advancements on the comprehension of the mechanisms sustaining the onset and progression of neurodegenerative diseases, the pathogenesis, and the molecular basis of these wide group of pathologies, characterized by the progressive dysfunction of specific populations of neurons accompanied by neuroinflammatory processes, are not yet fully understood. In this scenario, understanding both neuronal cell death pathways and the cross-talk between neurons and non-neuronal cells, not only provides knowledge of the pathobiology, but can also suggest novel therapeutic modalities against neurodegeneration. The interest concerning S100A4 in this topic is emerging in recent years, given the growing literature supporting its involvement in such processes.

Several studies demonstrated a neuroprotective role for S100A4. In support of this function, Pankratova and coauthors showed that S100A4 exerted a neuroprotective action that mainly involved the growth factor receptor ERBB4 and its ligand Neuregulin 1 (NRG), crucial players of neuronal plasticity through Ras-mitogen-activated protein kinase (MAPK) and AKT pathways [55]. It is known that NRG/ERBB4 axis controls synaptic transmission and neuronal excitability, stimulates neurogenesis, neuronal differentiation and survival, providing beneficial effects in models of Alzheimer’s (AD) and Parkinson’s diseases (PD), in cerebral ischemia, epilepsy, and schizophrenia [55,56]. In cellular models of neurodegeneration employing primary and immortalized dopaminergic neurons, S100A4 and its mimetic peptides exerted neuroprotective effects relying on ERBB4 expression and its partners ERBB2/AKT, suggesting their potential use as neuroprotectants in neurodegenerative diseases such as PD [55]. PD is characterized by dysfunctions of the substantia nigra, motor cortex and hippocampus, leading to motor impairments associated with cognitive and memory deficits in patients. High-frequency stimulation (HFS) of the ventrolateral thalamus was found to be an effective treatment in reducing typical tremors of PD disease, mainly due to the inactivation of the overactive thalamic cells [57,58]. By analyzing gene expression profiles in rat hippocampus, Huguet et al. showed that chronic HFS modulated the expression of 176 genes, mainly involved in neurogenesis and proliferation, including nestin and doublecortin, or in neural plasticity and migration, such as S100A4. S100A4 was one of the most significantly up-regulated genes after HFS [59], corroborating its involvement in neural plasticity [31,55]. Likewise, in the myelin protein zero null mice, a model of Charcot-Marie-Tooth type 1 disease, an inherited human demyelinating neuropathy that leads to a severe progressive loss of myelin and degeneration of both motor and sensory myelinated axons, chronic administration of the H3 peptide demonstrated a long-term neuroprotective effect. By mimicking the S100A4 activity, H3 reduced demyelination and axonal loss and improved overall nerve conduction [23].

Interestingly, besides exerting neuroprotective actions, S100A4 is involved in the inflammatory machinery that could contribute to the pathogenesis of nervous system disorders. It is well-known that chronic neuroinflammation mediated by microglia and astrocytes, a common feature across neurodegenerative disorders, often occurs before neuronal loss, participating in both the onset and progression of the diseases [60]. In this regard, as demonstrated in a recent study, the genome-wide analysis of differential expression, performed in several cell phenotypes, identified S100A4 as one of the 45 genes whose levels are increased in microglia derived from frontal and temporal cortices of patients with AD, compared to control individuals [40]. These data suggested a potential role for S100A4 in the inflammatory aspects of this type of dementia. This function has also been hypothesized in amyotrophic lateral sclerosis (ALS), a neurodegenerative disease characterized by a strong inflammatory component. Indeed, S100A4 was found to be significantly up-regulated in astrocytes and microglia in the spinal cord of a transgenic superoxide dismutase 1 (SOD1)-G93A animal model and in activated primary microglia [34]. The overexpression of S100A4 on activated glial cells found in ALS rodent models, together with the increase of vimentin and α-smooth muscular actin in the affected regions, suggested that in addition to its well-known functions in regulating inflammation and cell motility, S100A4 participates in the induction of reactive gliosis and in the formation of the so-called “glial scar” [61]. This glial response characterizes ALS-affected tissues and results from the massive migration of astrocytes and microglia toward the site of injury [62,63]. Remarkably, S100A4 over-expression in glial cells was detectable at the pre-symptomatic phase of the disease in SOD1-G93A animals [34], data in accordance with the S100A4 gene up-regulation previously shown in the astrocytes of pre-symptomatic SOD1-G37R ALS mice [64], implying that S100A4 could represent a candidate molecule involved in the early ALS pathogenic mechanisms. Recently, S100A4 was found increased in a different mouse model of ALS, associated with FUS gene, where S100A4 overexpression has been detected in both gray and white matter of diseased mice spinal cord (personal communication).

A proinflammatory role of S100A4 was further demonstrated in multiple sclerosis, a demyelinating neurodegenerative disease characterized by strong inflammatory and immune responses. Among a total of 1120 proteins analyzed in the spinal cord and in the brain of two mouse models of experimental autoimmune encephalomyelitis (EAE) during different stages of the disease, S100A4 was found as one of the 13 most significantly up-regulated proteins with functions linked to inflammation, leukocyte adhesion and migration and tissue repair. The authors found that S100A4 was strongly up-regulated in CNS-infiltrating CD4+ cells during EAE progression and, interestingly, its levels decreased with the improvement of the disease, suggesting therefore a key role for S100A4 in the inflammatory environment contributing to pathology, particularly by promoting the recruitment of inflammatory cells [65]. Accordingly, in a recent paper [66] integrative omics analysis revealed an up-regulation of S100A4 during neuronal differentiation and, remarkably, a high expression of S100A4, especially in hippocampal and cerebellar neurons, during the chronic progressive phases and in the inflammatory phase of EAE in mice, suggesting that S100A4 could be strongly induced by inflammation. Moreover, S100A4 levels were found to be significantly increased in the cerebrospinal fluid of patients with multiple sclerosis compared to neurologic controls [66], suggesting a possible use of S100A4 as a biomarker for the disease.

Very intriguingly, recent studies show that S100A4 is one of the 88 up-regulated genes of the pan-neurodegenerative signature resulting from the meta-analysis of human CNS transcriptomic datasets from 2,600 AD, Lewy body disease and ALS-frontotemporal dementia patients and age-matched controls, suggesting that S100A4 represents a common signature driving neurodegeneration [67,68].

### 3.3. Acute Injuries

The role of S100A4 was documented in nervous system acute injuries. The protein was found to be highly expressed in lesioned CNS and PNS, particularly in the glial component, i.e., in CNS astrocytes and PNS Schwann cells. The role of S100A4 in both controlling neuron survival and axonal elongation, as well as in the recruitment of inflammatory cells, was observed in neurological disorders such as brain trauma and excitotoxic damage as occurring in epilepsy. Brain injury is a multifaceted disease where the first harm leads to a secondary damage, characterized by inflammatory-related pathways that converge into neuronal death [69]. In both patients and experimental models of brain injury, S100A4 was overexpressed in WM astrocytes and in blood cells. It was demonstrated that in mice with cryogenic lesion, a brain insult generating a secondary response similar to that seen after severe head trauma, S100A4 increased in both the lesion site and in the contralateral WM, possibly due to the activation response of astrocytes following focal brain injuries. S100A4 expression was also increased, particularly in the hippocampus, in mice after kainic acid-evoked excitotoxicity, a type of brain injury adopted as a model of human temporal lobe epilepsy [70]. S100A4 neuroprotective role in the aforementioned types of injuries was established by the results obtained by genetic ablation. In fact, the lack of S100A4 in mice aggravated neuronal loss, as detected by the increased number of neurons undergoing oxidative stress. However, S100A4 deletion did not affect astrocytosis, microgliosis, and the extent of axonal and oligodendrocyte loss in the lesioned areas. Finally, the S100A4 neuroprotective role in these models of brain injury was confirmed by the use of synthetic peptides that contained motifs belonging to S100A4 [70]. Consistently with these results, in a rat model of traumatic brain injury, the analysis of the gene expression signature aimed to find novel treatments able to modulate both pathologic or protective genes, has showed S100A4 as one of the most significantly altered genes in the perilesional cortex and in the thalamus after damage. Moreover, S100A4 was one of the target genes modified by different drugs proposed as recovery-enhancing treatments in brain damage [71].

The neuroprotective effect of S100A4 was finally demonstrated in PNS injury, where S100A4 peptide mimetic H3 has shown electrophysiological, behavioral, and morphological recovery, in an in vivo model of sciatic nerve crush; in vitro both S100A4 and H3 elicited neurite branching. On the basis of these results, S100A4 was proposed to take a central role to improve initial axonal sprouting and integrity after nerve transection and reconstruction [23]. Recent evidence indicates a neuroprotective effect of S100A4 after retinal ischemia-reperfusion injury in mice, where its overexpression inhibits apoptosis in retinal ganglion cells by activating the AKT pathway [72].

Notwithstanding the neuroprotective effects of S100A4 in acute brain stress [70], it was observed the protein represents a specific marker for WM astrocytes [21] and can influence the formation of a “non-permissive” glial scar following nerve injuries, therefore limiting axonal regeneration. In this aspect, S100A4 was found markedly overexpressed mainly in WM astrocytes adjacent to the site of transection in the spinal cord after sciatic nerve or dorsal root resection [21,24], suggesting its participation in the formation of a “non-permissive” environment [24]. It is well-known that WM astrocytes are key players in the development of a glial scar that appears following injury and that represents a major limit for efficient axonal growth and remyelination by oligodendrocytes [73,74]. Likewise, it was reported that spinal cord transection induced sustained S100A4 mRNA and protein expression in astrocytes in the rostral and caudal areas near the injury site, with increased levels observed up to 28 days post-injury, indicating a role of this molecule in long-term astrocytic responses after damage [75]. Moreover, proximally and distally to the injury site an evident increase of S100A4 has been detected also in satellite cells in the ganglion and in Schwann cells surrounding the injured axons, after peripheral nerve or dorsal root injury [22]. To clarify the contribution of S100A4 to the formation of a glial scar and its role in supporting the regeneration of sensory axons, Trolle and coauthors administered boundary cap neural crest stem cells, known to support the growth of sensory axons during development, to a murine model of dorsal root injury. They demonstrated that the stem cells, although forming permissive gaps in the glial scar, differentiated into non-permissive S100A4-expressing astrocytes that did not contribute to the regeneration of sensory axons [24]. These data confirmed the induction of high levels of S100A4, particularly in WM astrocytes after sciatic nerve or dorsal root injury [35,76] and indicated a role for S100A4 in the formation of glia scar after injury in the spinal cord. In line with these observations, in S100A4 null mice, the injection of ethidium bromide, employed as a demyelinating agent, induced astrocyte migration into the demyelinated area. This event was not observed in non-transgenic mice, where hypertrophic, S100A4-positive astrocytes remaining at the injury site boarder, formed a robust glial scar. These results indicated that S100A4, by reducing the migratory ability of reactive WM astrocytes in the CNS lesion, was strongly involved in the establishment of fibrotic non-permissive environment after damage [38].

## 4. Conclusions

Mounting evidence indicates that S100A4 can influence the properties of several cell types present in the nervous system in physiological and pathological conditions [8]. By affecting the functions of astrocytes, microglia, infiltrating cells and neurons, S100A4 regulates inflammation and immune reactions and modulates neuronal plasticity and survival.

**Figure 2:**
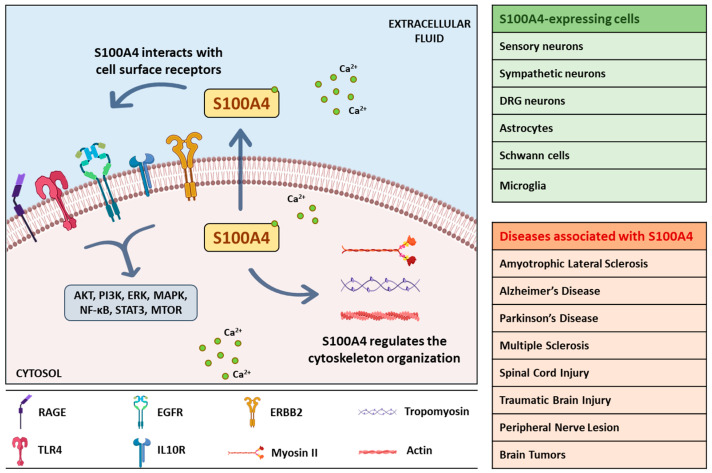
Schematic illustration of intracellular and extracellular actions of S100A4. Cytosolic S100A4 binds to calcium and interacts with cytoskeletal proteins; secreted S100A4 interacts with cell surface receptors leading to downstream signaling. Neuronal and non-neuronal cells expressing S100A4 and the nervous system pathologies involving S100A4 are shown in the box. AKT, protein kinase B; EGFR, epidermal growth factor receptor; ERBB2, Erb-B2 Receptor Tyrosine Kinase 2; ERK, extracellular signal-regulated kinase; IL10R, interleukin 10 receptor; MAPK, mitogen-activated protein kinase; MTOR, mammalian target of rapamycin; NF-κB, nuclear factor-κB; PI3K, phosphoinositide 3-kinases; RAGE, receptor for advanced glycation end products; STAT3, signal transducer and activator of transcription 3; TLR4, Toll Like Receptor 4.

In this review, we summarized the actions of S100A4 in the nervous system physiopathology (Figure 2) highlighting its involvement in molecular pathways critical for disease development and of potential interest for clinical applications.

According to the context, S100A4 can elicit compounding effects, and should therefore be taken into consideration in strategies of intervention for multifactorial non-cell autonomous diseases as those represented by neurological disorders.

As with what occurs in fibrotic processes, where S100A4 can be either degenerative or regenerative [11], S100A4 can sustain both regenerative and degenerative mechanisms in the nervous system, acting as a neuroprotectant and as a pro-inflammatory and pro-fibrotic molecule, limiting the rescue of injured axons. The role of S100A4 depends on the specific context affecting the nervous system, e.g., acute or chronic injury, CNS or PNS areas, the presence or absence of immune/pro-inflammatory cells, on the amount of its release and on its oligomerization extent. Further studies are necessary to gain more insight to establish the significance of S100A4 up-regulation in different cell types and to unveil how S100A4 modulation affects the outcome of nervous system pathologies. To date, S100A4 could be included among the potential molecules capable of reducing or preventing neuronal damage, since synthetic peptides mimicking S100A4 exert neuroprotective effects in models of acute brain injuries [23,55,70]. Nonetheless, S100A4 inhibitors, particularly by reducing glial hyperactivation following acute or chronic damage, could be used to ameliorate neuroinflammatory mechanisms in neurological diseases. In this regard, niclosamide, a transcriptional inhibitor of S100A4, undergoing repurposing for numerous inflammatory and fibrotic diseases [77,78,79], has shown beneficial effects in diminishing gliosis and axonal impairment in a model of ALS, and in improving peripheral neuropathy [80], representing a promising drug for treating nervous system diseases. Altogether, these findings strongly suggest that S100A4 modulation can affect the mechanisms involved in nervous system disorders, and we therefore believe that the investigation of S100A4-related drugs in pre-clinical and clinical trials could be attractive to the field of neurobiology.

## Data Availability

Not applicable.
